# Supplementary material for: Mid and hindgut transcriptome profiling analysis of Atlantic salmon (Salmon salar) under unpredictable chronic stress
Source: R Soc Open Sci. 2020 Feb 26;7(2):191480. doi: 10.1098/rsos.191480 (PMC7062075; doi:10.1098/rsos.191480)
Supplement: Supplementary figures 1-12 [file rsos191480supp1.pdf]

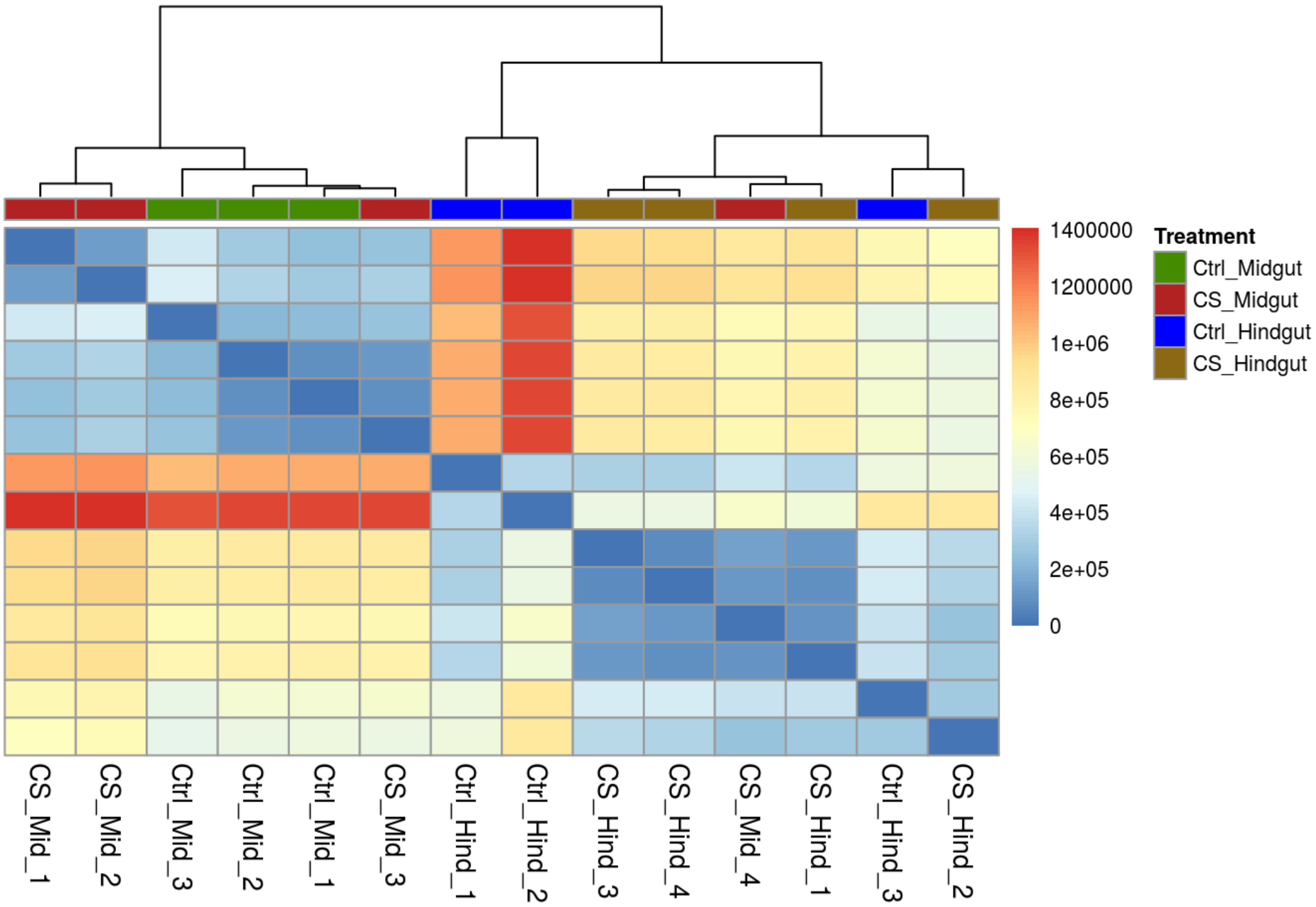


**Supplementary figure 1**. Hierarchical clustering of samples by read counts per gene. Heatmap and dendrogram. Heatmap legend and colours indicate between-sample Euclidean distance. Note that both axes of the heatmap represent the list of samples, but the sample labels on the x axis

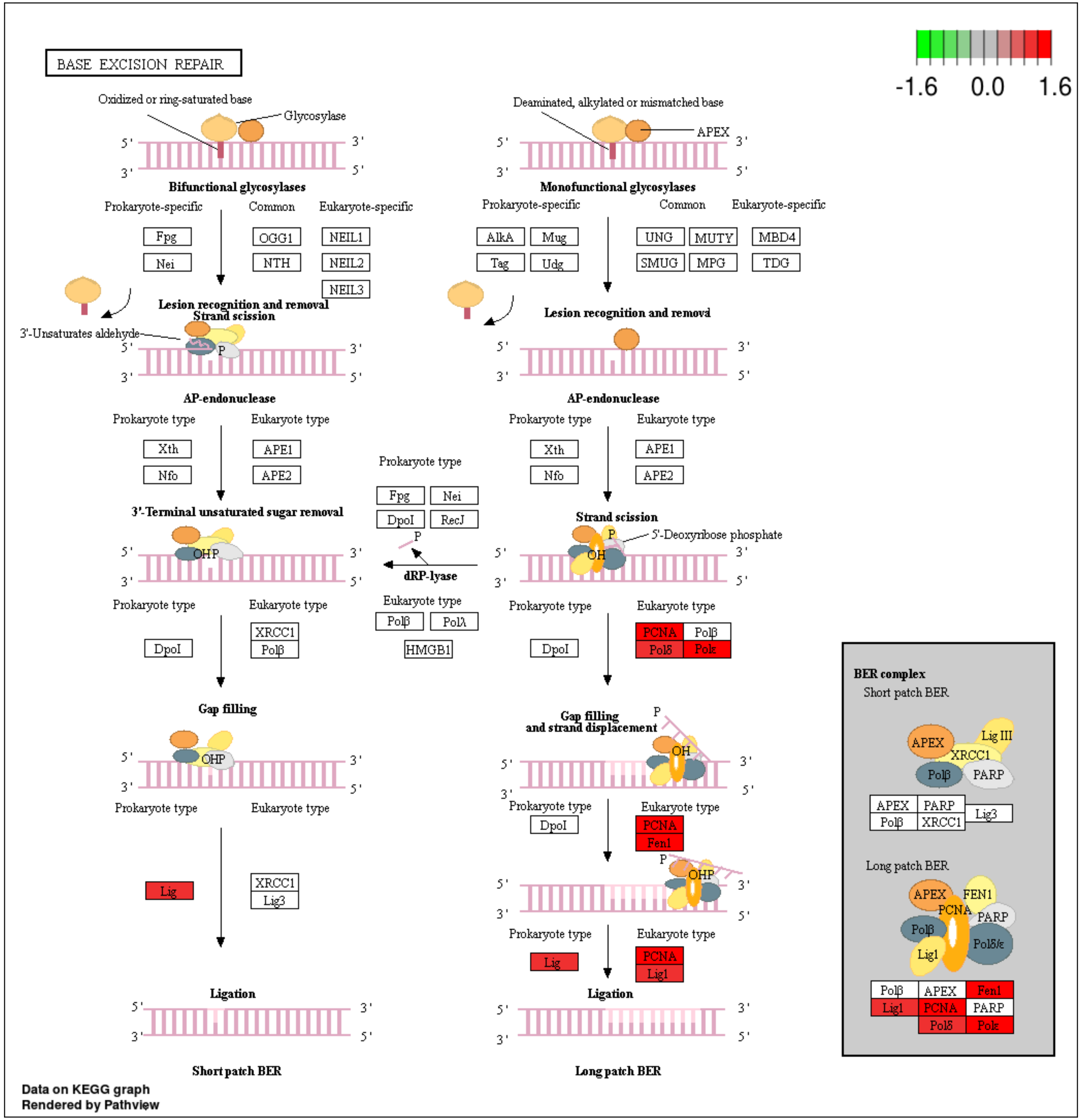


**Supplementary figure 2**. Metabolic pathway map showing differentially expressed genes within base excision repair for midgut control vs UCS. Red colored genes are upregulated and green colored genes are downregulated

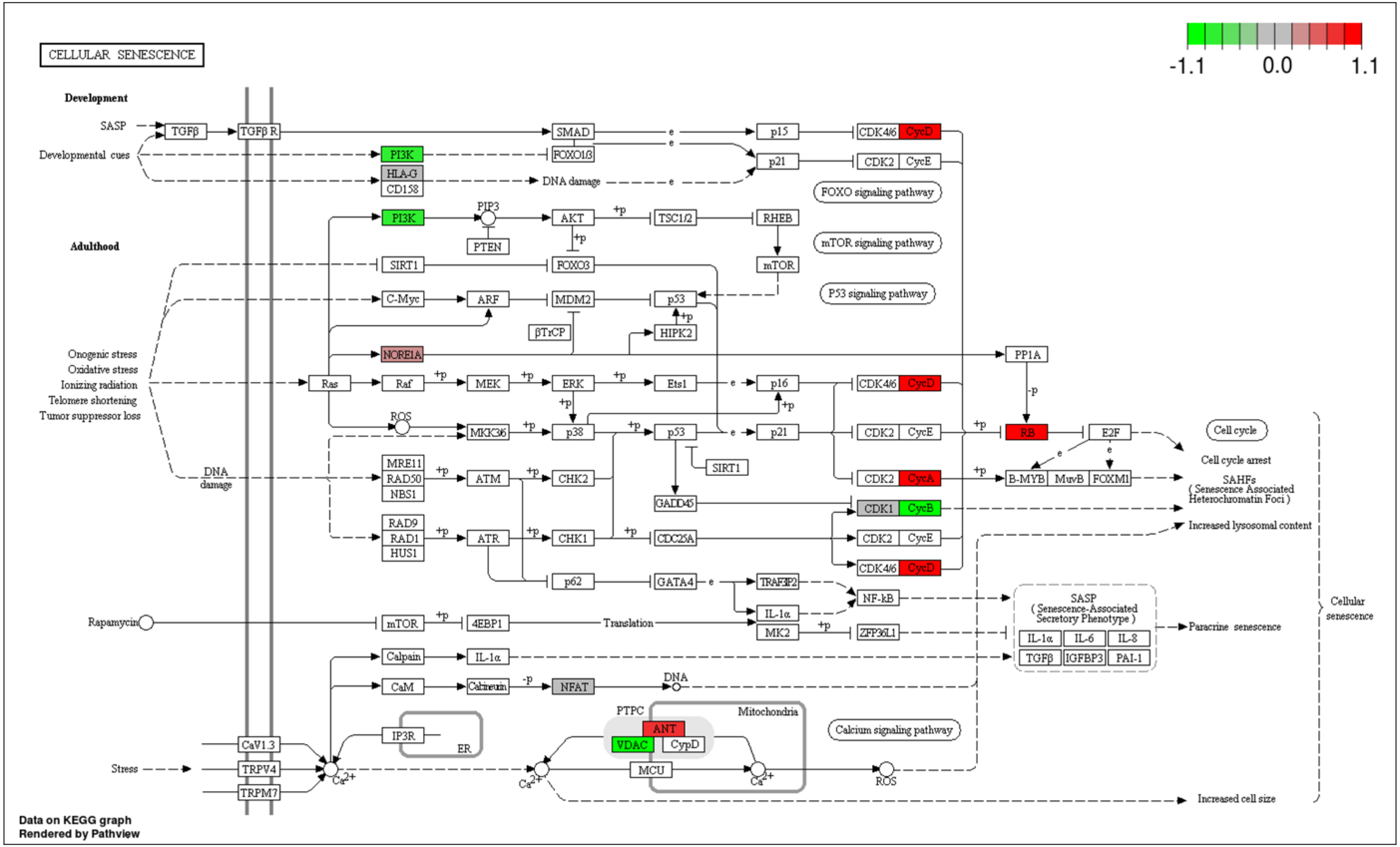


**Supplementary figure 3**. Metabolic pathway map showing differentially expressed genes within cellular senscene for hindgut control vs UCS. Red colored genes are upregulated and green colored genes are downregulated

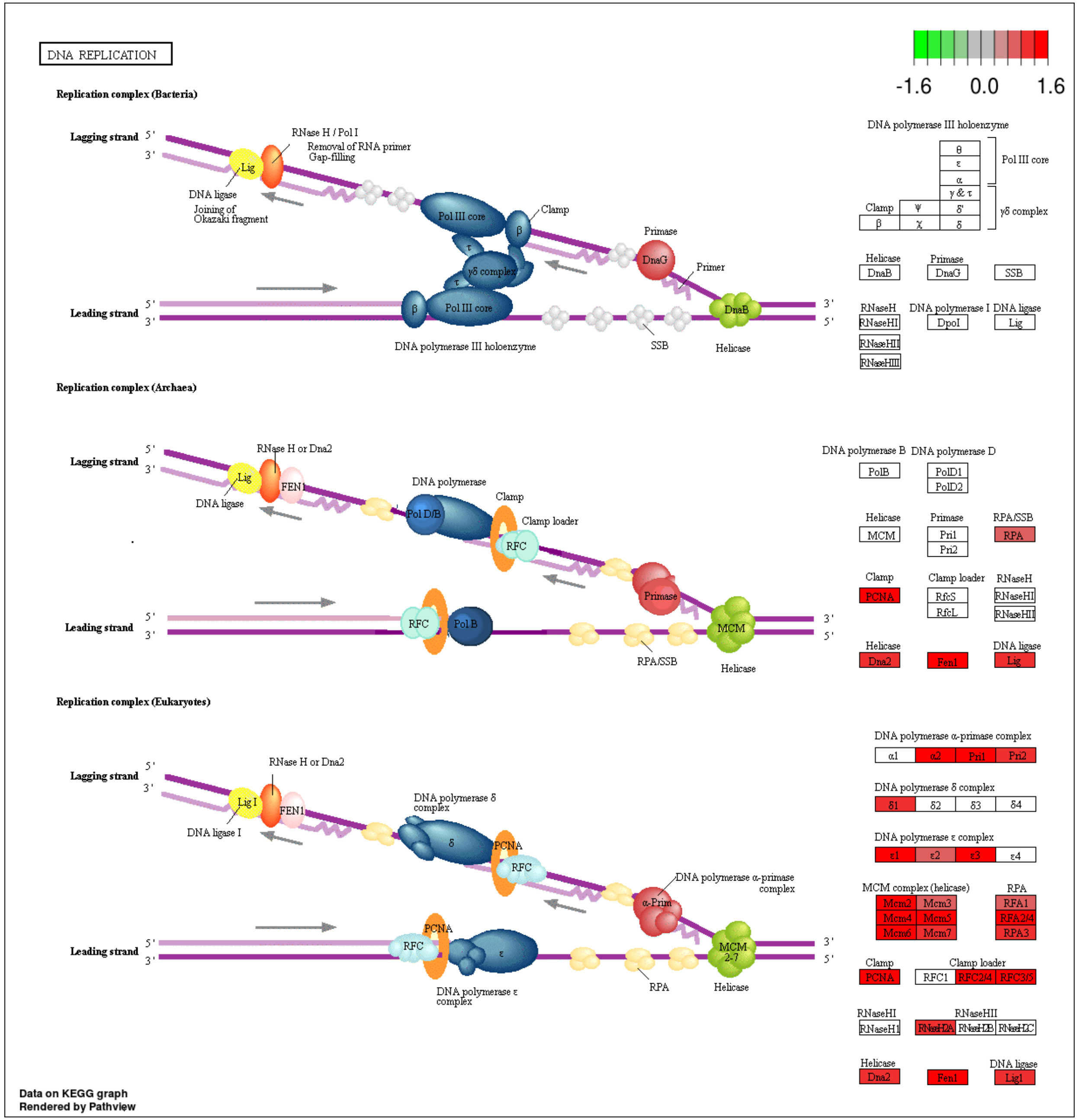


**Supplementary figure 4**. Metabolic pathway map showing differentially expressed genes within DNA replication for midgut control vs UCS. Red colored genes are upregulated and green colored genes are downregulated

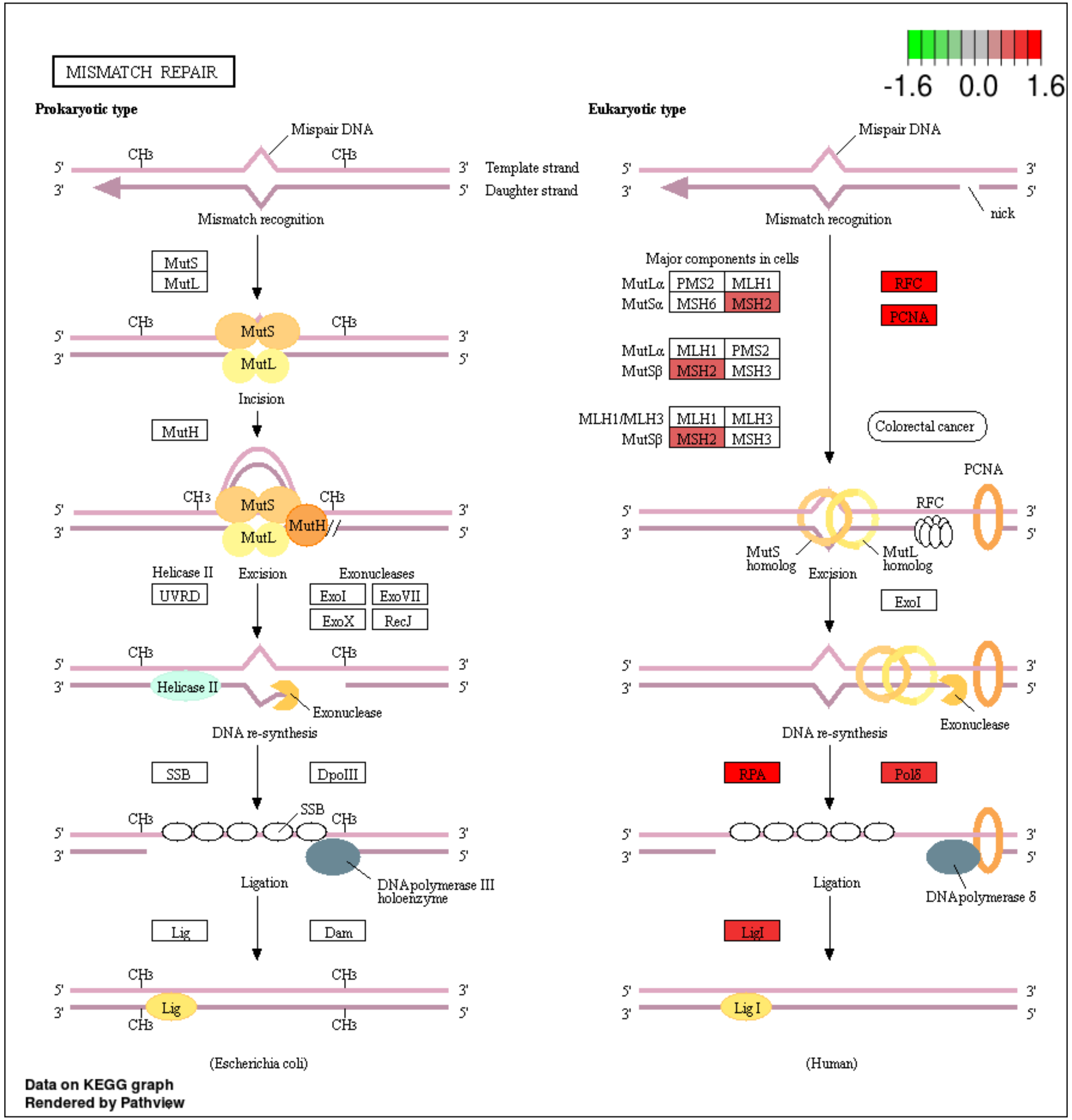


**Supplementary figure 5**. Metabolic pathway map showing differentially expressed genes within mismatch repair for midgut control vs UCS. Red colored genes are upregulated and green colored genes are downregulated

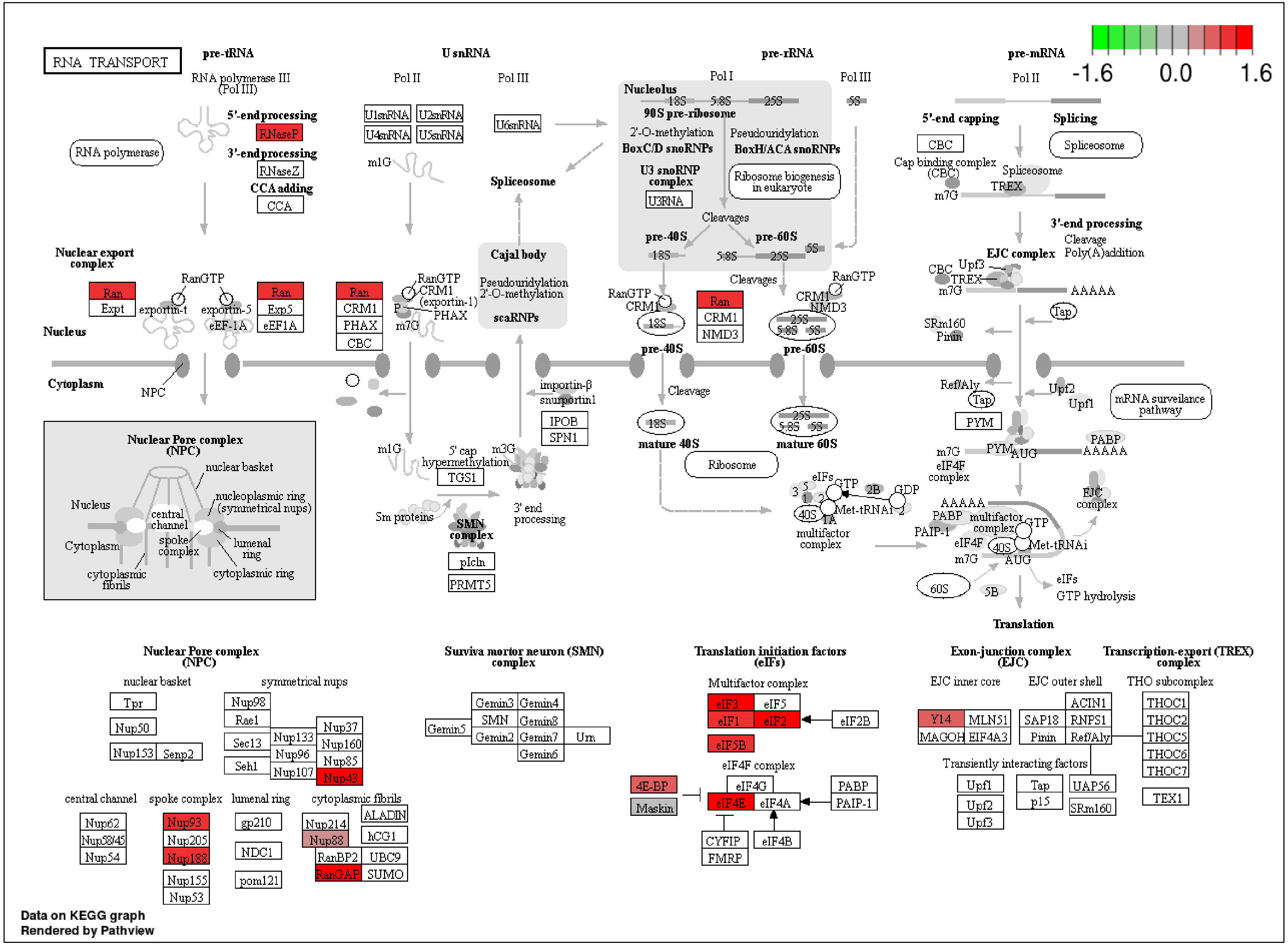


**Supplementary figure 6**. Metabolic pathway map showing differentially expressed genes within RNA transport for midgut control vs UCS. Red colored genes are upregulated and green colored genes are downregulated

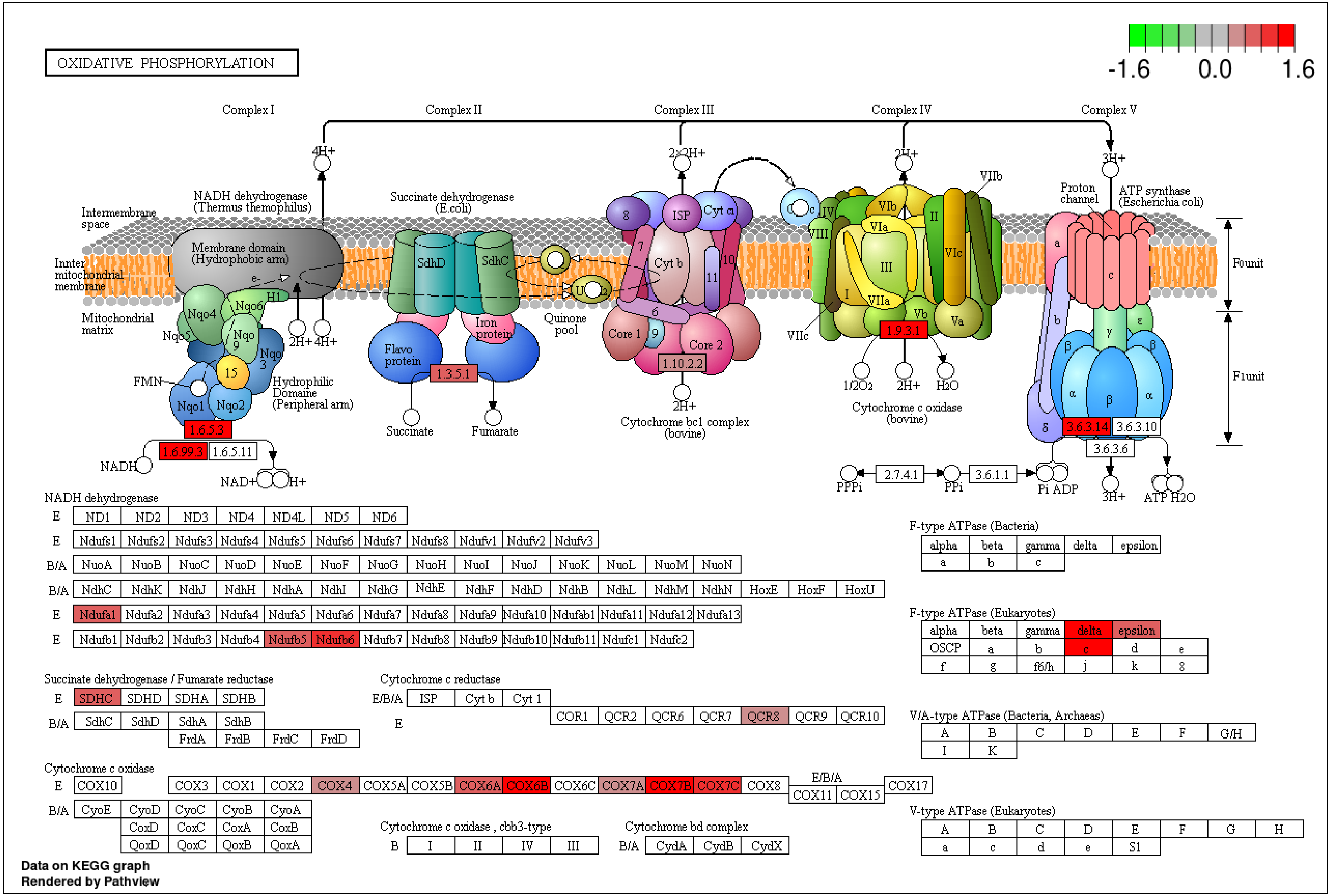


**Supplementary figure 7.** Metabolic pathway map showing differentially expressed genes within oxidative phosphorylation for midgut control vs UCS. Red colored genes are upregulated and green colored genes are downregulated

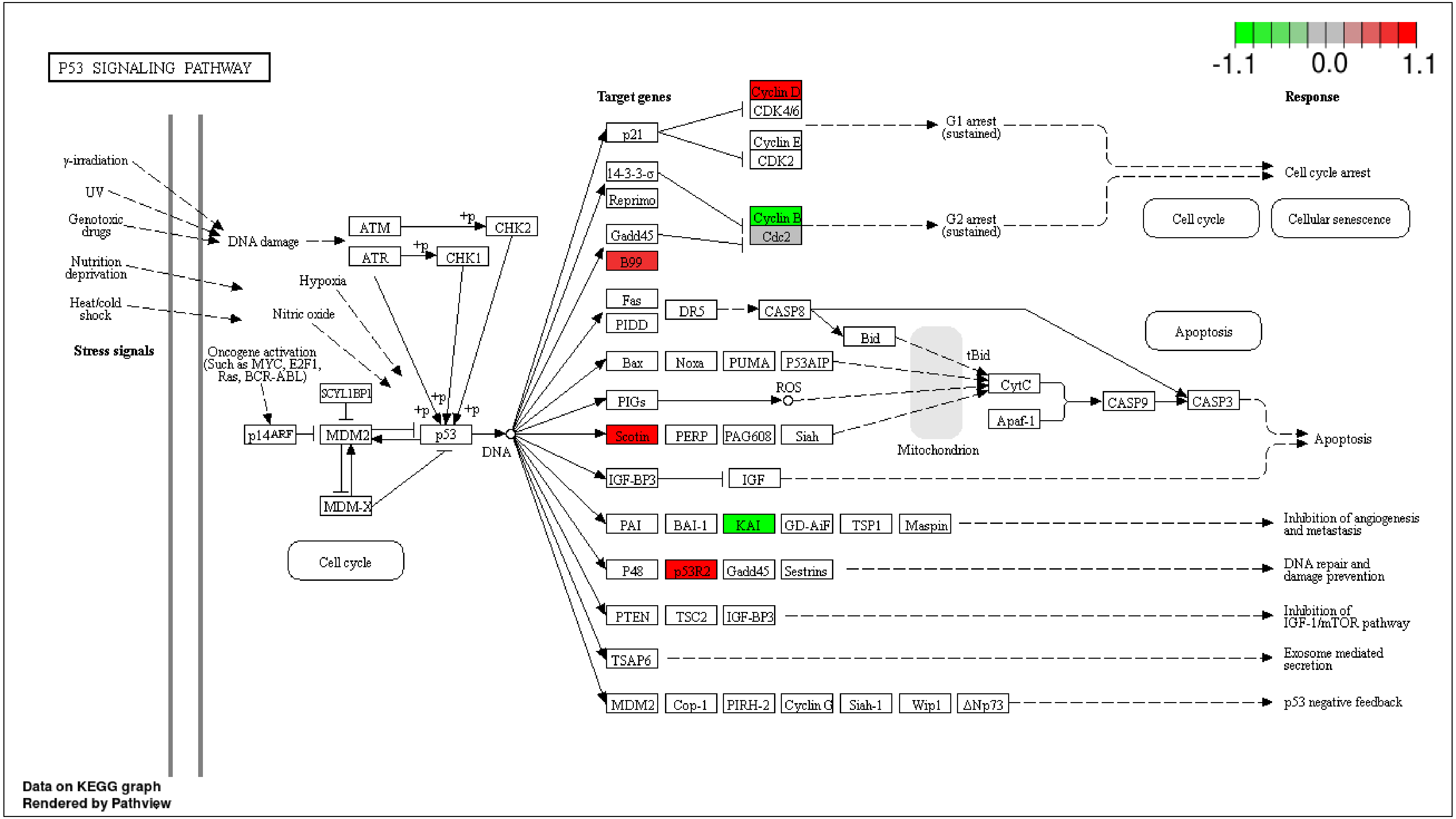


**Supplementary figure 8.** Metabolic pathway map showing differentially expressed genes within P53 signalling pathway for hindgut control vs UCS. Red colored genes are upregulated and green colored genes are downregulated

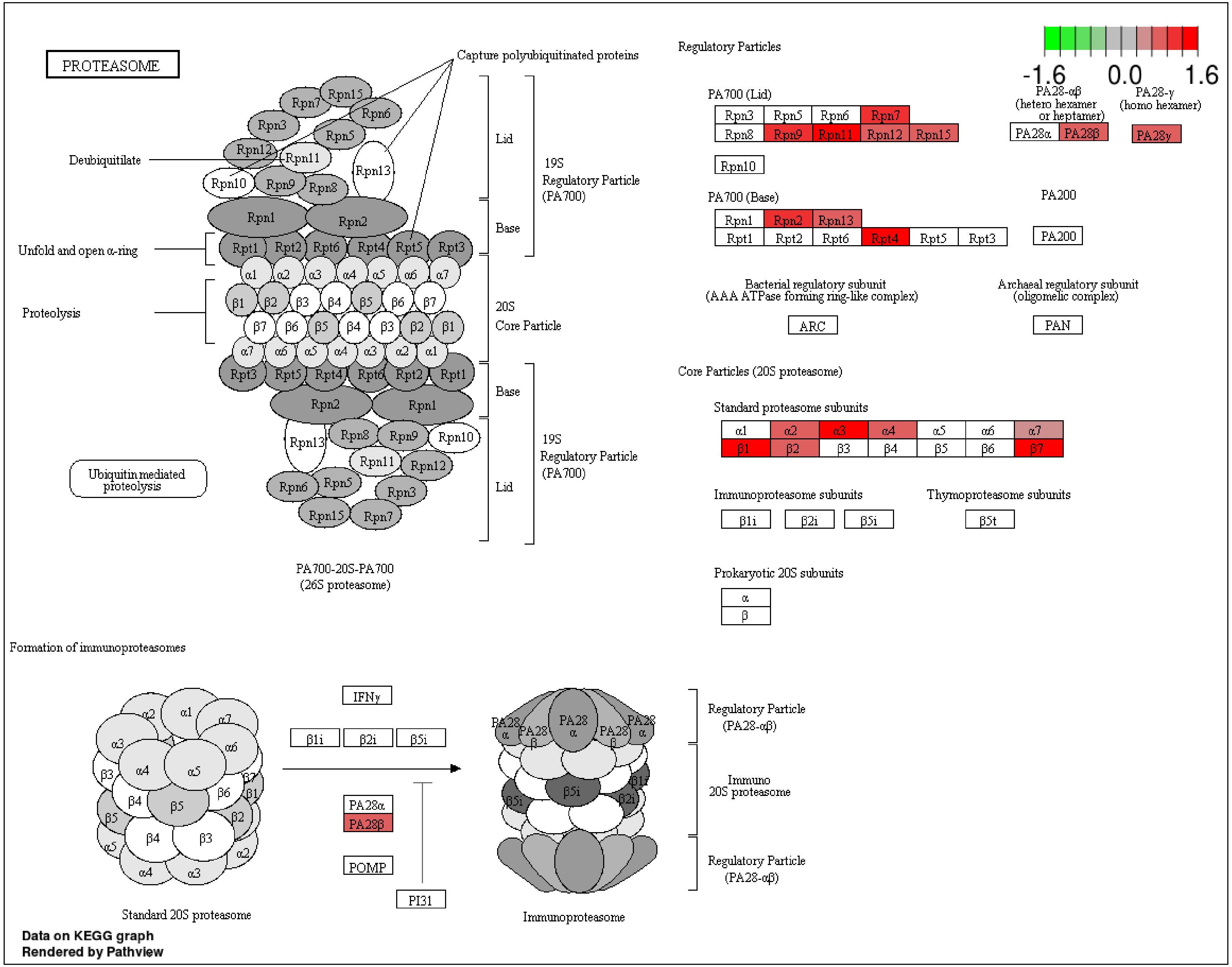


**Supplementary figure 9.** Metabolic pathway map showing differentially expressed genes within the proteasome complex for midgut control vs UCS. Red colored genes are upregulated and green colored genes are downregulated

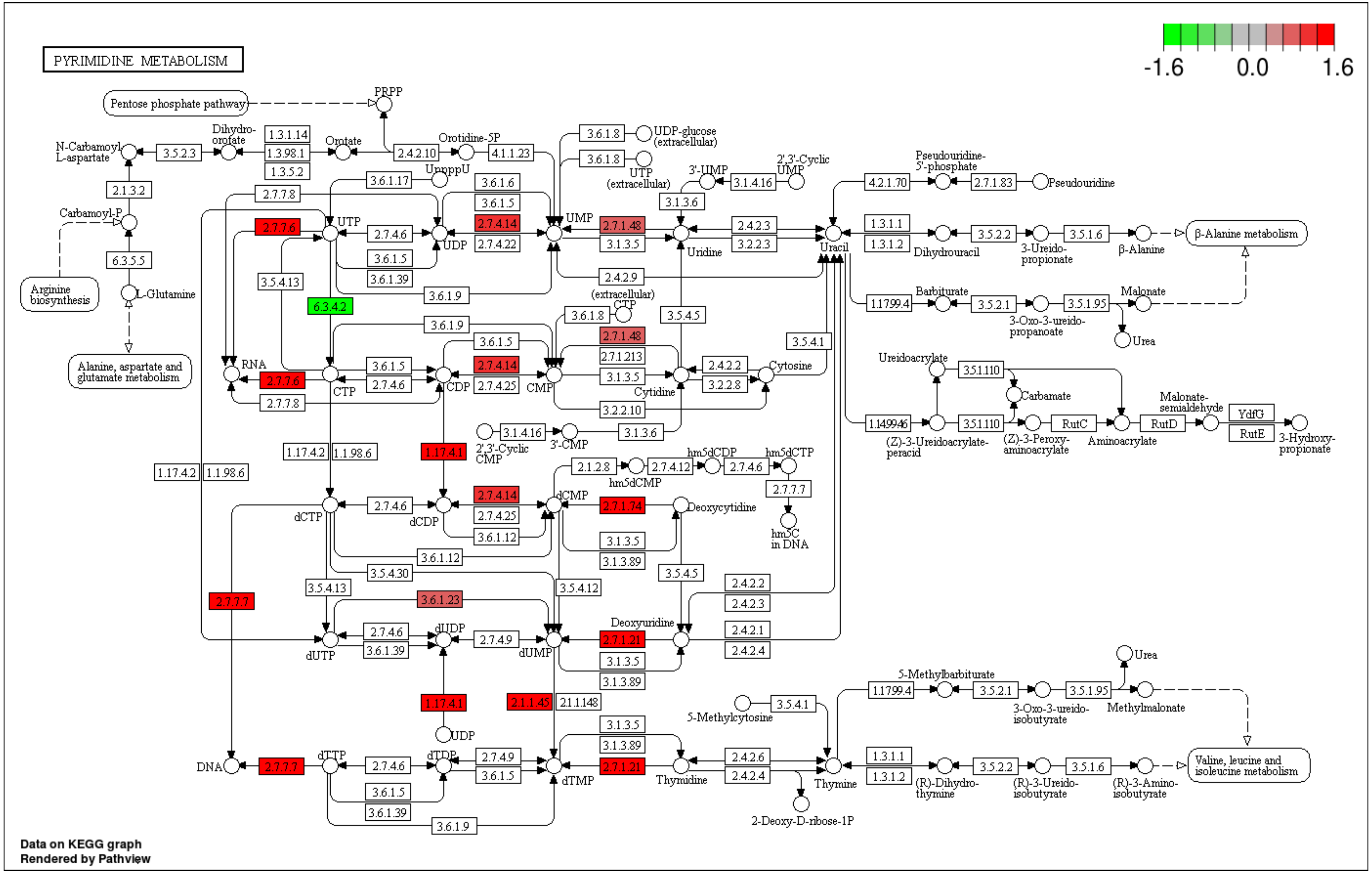


**Supplementary figure 10**. Metabolic pathway map showing differentially expressed genes within pyrimidine metabolism for midgut control vs UCS. Red colored genes are upregulated and green colored genes are downregulated

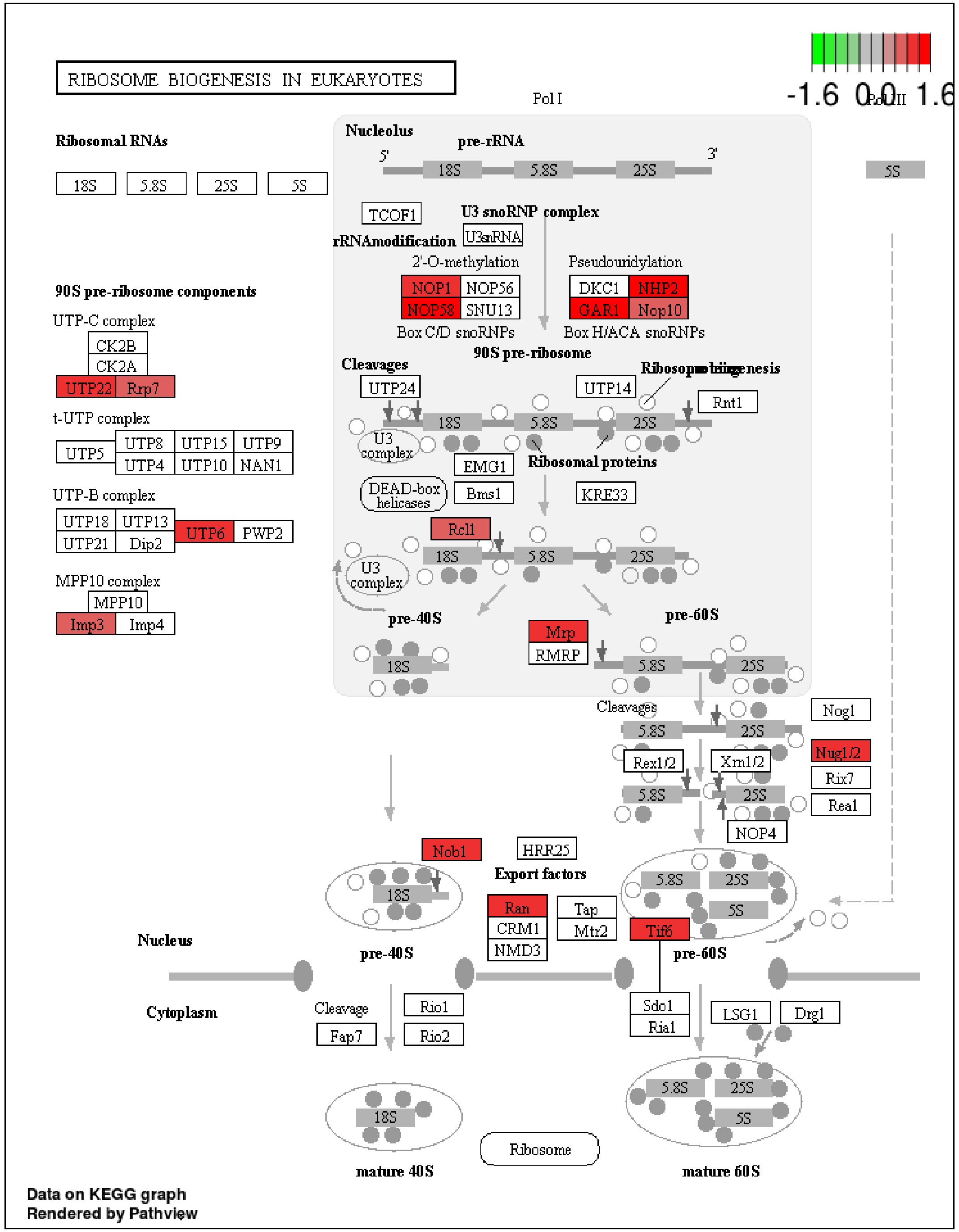


**Supplementary figure 11.** Metabolic pathway map showing differentially expressed genes within ribosome biogenesis in eucaryotes for midgut control vs UCS. Red colored genes are upregulated and green colored genes are downregulated

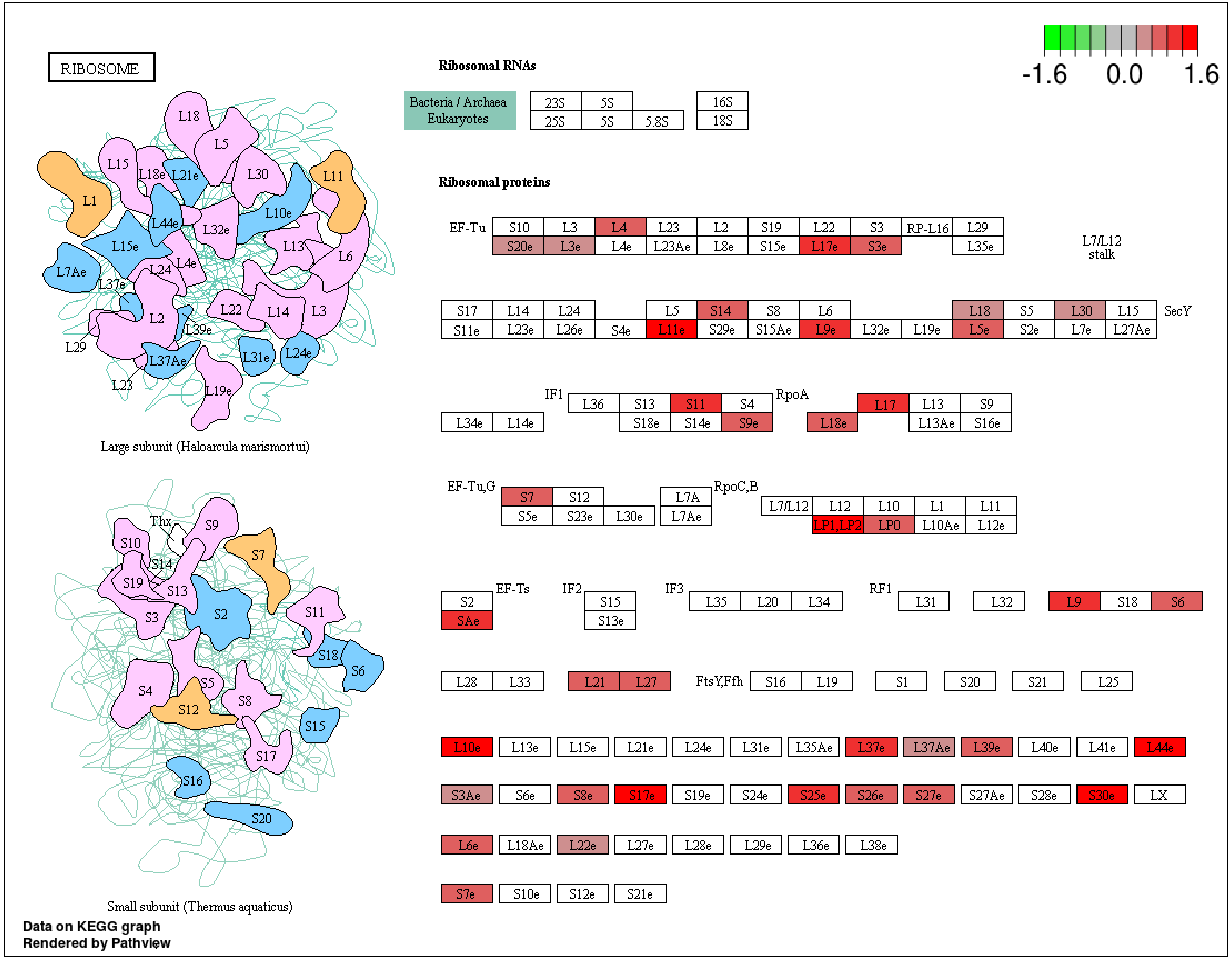


**Supplementary figure 12**. Metabolic pathway map showing differentially expressed genes within ribosome complex for midgut control vs UCS. Red colored genes are upregulated and green colored genes are downregulated
