# Supplementary material for: Mid and hindgut transcriptome profiling analysis of Atlantic salmon (Salmon salar) under unpredictable chronic stress
Source: R Soc Open Sci. 2020 Feb 26;7(2):191480. doi: 10.1098/rsos.191480 (PMC7062075; doi:10.1098/rsos.191480)
Supplement: Supplementary figures 13-15 [file rsos191480supp2.pdf]

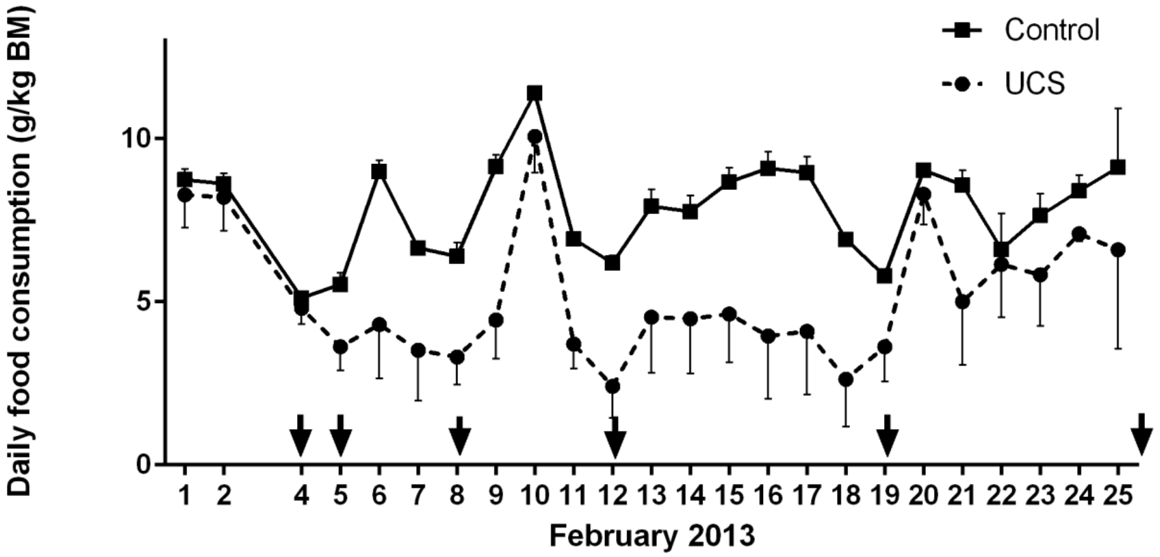

Figure 13. Daily food consumption during the experiment. The experiment started on February 4th and the last sampling was done on February 25th, sampling days are indicated by arrows. Food consumption is seen in the graph as food mass relative to body mass of the fish for control and UCS fish. Each point represents the ±s.e.m of the three replicate tanks. Modified from Madaro et al. 2015

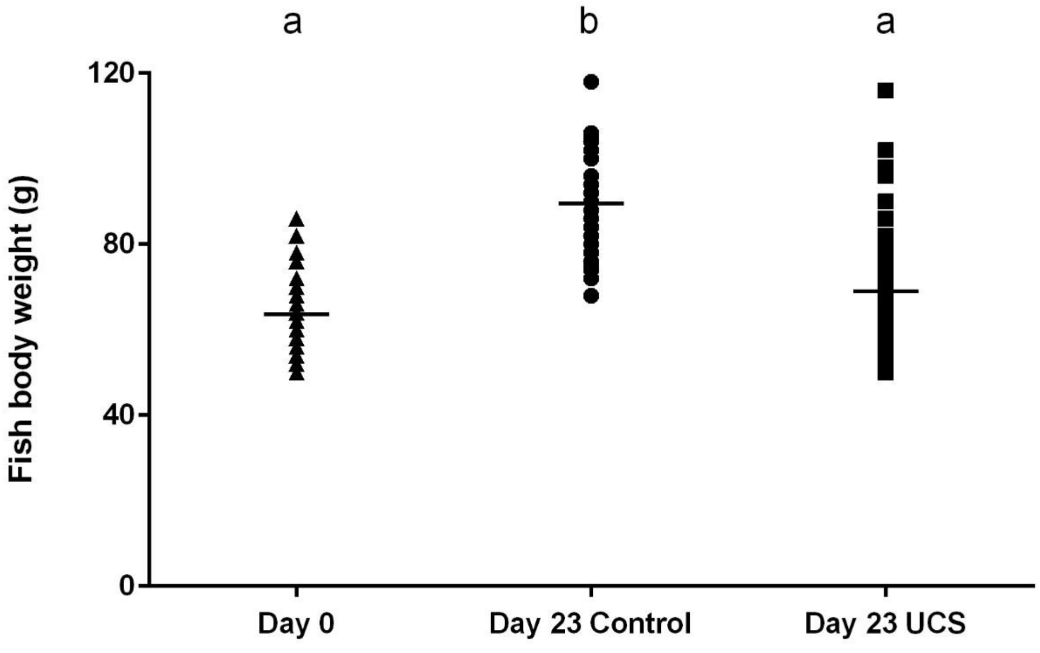

Figure 14. Fish body weight at day 0 and day 23 for control and UCS fish. N=45, 60, and 71 for day 0, control and UCS, respectively. Significant difference is indicated with different letters, calculated one-way ANOVA followed by Sidak's post hoc test; $P<0.025$. Modified from Madaro et al. 2015.

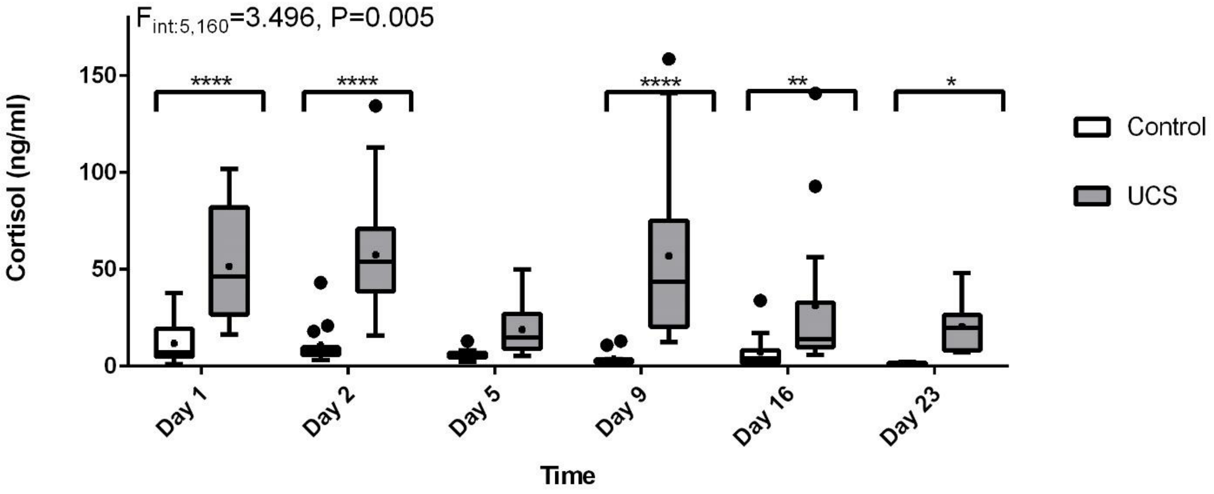

Figure 15. Plasma cortisol level of control and UCS fish were sampled at day 1, 2, 5, 9, 16, and 23. Two-way ANOVA analysis revealed a significant interaction effect (chronic stress×day of the experiment): $F_{5,160}=3.496$, $P=0.005$. Asterisk indicate the degree of significance (****P<0.0001, **P<0.01) between the two groups at the day of sampling, as calculated by Fisher's LSD *post hoc* test. Modified from Madaro et al. 2015
